# Age of puberty and Sleep duration: Observational and Mendelian randomization study

**DOI:** 10.1038/s41598-020-59811-9

**Published:** 2020-02-21

**Authors:** Jiao Wang, Man Ki Kwok, Shiu Lun Au Yeung, Jie Zhao, Albert Martin Li, Hugh Simon Lam, Gabriel Matthew Leung, Catherine Mary Schooling

**Affiliations:** 1 School of Public Health, Li Ka Shing Faculty of Medicine, The University of Hong Kong, Hong Kong, China; 2 Department of Pediatrics, Faculty of Medicine, The Chinese University of Hong Kong, Hong Kong SAR, China; 3 City University of New York, School of Public Health and Health Policy, New York, USA

**Keywords:** Epigenetics, Paediatric research

## Abstract

Earlier age of puberty has detrimental consequences for many aspects of health. Here, for the first time, we assessed the association of earlier puberty with sleep duration observationally and with validation using Mendelian Randomization. In the “Children of 1997” birth cohort (n = 8,327), we used adjusted multivariable logistic regression to assess the associations of each clinically assessed marker of earlier puberty with self-report sleep duration in adolescence. Using two-sample MR, we assessed the effect of earlier puberty timing based on 203 single nucleotide polymorphisms applied to genome wide association studies of sleep duration in adults (n = 335,410). In “Children of 1997”, cross-sectionally, older age of menarche was associated with longer (9+ hours) sleep duration [odds ratio (OR) 1.11, 95% confidence interval (CI) 1.01 to 1.21] at 13.5 years. The other earlier puberty markers were unrelated to sleep duration. Using inverse variance weighting, later of age at menarche increased adult sleep duration [0.020 per category, 95% CI 0.006 to 0.034]. This study demonstrated a causal effect of age at menarche on adult sleep duration, since age of menarche also affects obesity, our novel finding may be relevant to the observed relation of sleep duration with obesity and poor health.

## Introduction

Sleep serves an important restorative function in humans. However, sleep duration has decreased for children and adolescents in the last 20 years^1,2^. Short sleep duration is associated with a wide range of poor health outcomes including stress^3^, headache^4^, cardiovascular disease^5^, diabetes mellitus^6^ and even cancer^7^. Short sleep is also associated with screen time^8^, low socioeconomic position (SEP)^9^ and sedentary time^10^, making it difficult to ascertain whether these associations are causal or confounded. Sleep patterns change significantly at puberty with marked differences by sex^11,12^, suggesting puberty may be a critical stage for the development of sleep patterns. Earlier puberty is associated with many non-communicable diseases^13^. However a  Mendelian Randomization study was  not completely supportive of sleep as causing all  these outcomes^14^. As such sleep may also  be a consequence of other attributes, such as pubertal timing, which have causal effects on some non-communicable diseases. Notably, puberty affecting sleep duration falls within the emerging paradigm of evolutionary public health, i.e., considering health within the well-established paradigm of natural selection favoring reproductive success rather than wellbeing^15^. Laboratory studies suggested sex hormones affect sleep, potentially with sex-specific consequences. For instance, a randomized controlled trial (RCT) found testosterone shortened sleep duration in older men^16^, while estrogen plus progestin has been associated with better sleep in postmenopausal women^17^.

Observationally earlier menarche is associated with sleep disorders in adolescents from Western and Chinese settings^18,19^. However, observational studies are hard to interpret because they could be confounded by factors such as socio-economic position (SEP) and lifestyle. In this situation where experimental evidence is lacking Mendelian Randomization (MR), i.e., instrumental variable analysis with genetic instruments, may provide a way forward. Given genetic variants are allocated  randomly at conception, MR, as a quasi-experimental study design, is less susceptible to confounding and so provides an alternative means of assessing the causal effect of puberty on sleep duration. Several studies using this approach have recently clarified the associations of age of menarche with adolescent depression^20^, time spent in education^21^ and adult body mass index^13^, but no MR study has assessed the causal effects of earlier puberty on adult sleep duration^14^.

To clarify the effect of pubertal timing on sleep duration, we first conducted an observational study to assess associations of clinically assessed age of puberty with self-reported longer (9+ hours) compared to shorter (<9 hours) sleep duration in the large population-representative Hong Kong Chinese “Children of 1997” birth cohort. Second, we used two-sample MR to validate the causal effect of later puberty on adult sleep duration.

## Results

### Observational study in the Chinese “Children of 1997” birth cohort

Among the original 8,327 “Children of 1997” participants^22^, at the time of survey I (2008–09), 26 participants had permanently withdrawn, and 365 were not contactable. Of the 7,936 potential respondents, 3,603 provided sleep duration in Survey I (37.8% <9 hours and 62.2% 9+ hours), 3,933 provided sleep duration in Survey II (65.0% <9 hours and 35% 9+ hours) and 3,142 in the “Children of 1997” Biobank Clinical follow-up (79.0% <9 hours and 21% 9+ hours). In total 4,958 had age of menarche or voice breaking. The participants with and without puberty status differed in sex, parents’ birthplace, highest parental occupation, household income per head in quintiles and highest parental education levels but the Cohen effect sizes indicated these differences were small (Appendix Table 1). Multicollinearity is also  not a major issue in our study (Appendix Table 2). Mean age of onset of breast development was 9.6 years and 10.8 years for genitalia development. Girls (10.56 ± 1.03) had earlier age of onset of pubic hair than boys (11.48 ± 1.09). Mean age of menarche was 11.9 years and mean age of voice breaking was 13.1 years. Later pubertal development was associated with higher family SEP, such as parents’ birthplace, highest parental occupation, household income per head in quintiles and highest parental education level (Table 1).

**Table 1:** Baseline Characteristics according to Age of Puberty from Hong Kong’s “Children of 1997” Birth Cohort. (Available case Analysis).

Characteristic	Classification	n	Onset of Breast/Genitalia Mean (SD), y	P value	n	Onset of Pubic Hair Development Mean (SD), y	P value	n	Age of Menarche/Voice breaking, Mean (SD), y	P value
Gender				<0.01			<0.01			<0.01
Boys		1921	10.88 (1.08)		1428	11.48 (1.09)		1781	13.08 (1.18)	
Girls		2961	9.60 (1.28)		1736	10.56 (1.03)		3177	11.94 (1.08)	
Parents’ birthplace				<0.01			<0.01			0.109
Both parents migrant		1156	10.00 (1.33)		716	10.86 (1.07)		1244	12.37 (1.26)	
One parent migrant		980	10.03 (1.42)		638	10.90 (1.16)		1055	12.30 (1.23)	
Both parents Hong Kong		2608	10.18 (1.34)		1717	11.06 (1.17)		2555	12.39 (1.25)	
Highest parental occupation				<0.01			0.02			<0.01
I (professional)		1046	10.2 (1.38)		691	11.06 (1.20)		1089	12.45 (1.26)	
II (managerial)		625	10.05 (1.30)		403	11.00 (1.14)		639	12.33 (1.24)	
IIINM (nonmanual skilled)		1298	10.14 (1.39)		855	10.97 (1.16)		1262	12.34 (1.21)	
IIIM (manual skilled)		700	10.03 (1.36)		454	10.94 (1.11)		748	12.35 (1.26)	
IV (semi-skilled)		404	9.96 (1.28)		261	10.87 (1.17)		420	12.26 (1.22)	
V (unskilled)		155	9.95 (1.30)		99	10.98 (1.08)		141	12.29 (1.30)	
Household income per head in quintiles				0.04			0.01			0.03
1st quintile (HK$ 1751 ± 413)		806	10.03 (1.38)		519	10.87 (1.11)		835	12.26 (1.23)	
2nd quintile (HK$ 2856 ± 325)		899	10.09 (1.40)		570	10.92 (1.21)		868	12.34 (1.27)	
3rd quintile (HK$ 4362 ± 556)		845	10.06 (1.33)		555	10.99 (1.12)		897	12.38 (1.22)	
4th quintile (HK$ 6822 ± 886)		901	10.16 (1.35)		616	11.06 (1.14)		892	12.36 (1.24)	
5th quintile (HK$ 14850 ± 16050)		900	10.21 (1.34)		564	11.07 (1.22)		908	12.45 (1.23)	
Highest parental education level				<0.01			<0.01			<0.01
Grade 9 or below		1415	10.02 (1.37)		894	10.90 (1.12)		1457	12.25 (1.25)	
Grade 10–11		2115	10.12 (1.35)		1397	10.96 (1.13)		2121	12.40 (1.25)	
Grade 12 or above		1286	10.17 (1.37)		831	11.08 (1.21)		1333	12.41 (1.23)	

Age of menarche was positively associated with sleep duration [odds ratio (OR) 1.11, 95% CI (1.01 to 1.21)] at age 13.5 years, but other measures of puberty timing were unrelated to sleep duration. (Table 2).

**Table 2:** Adjusted* Associations of Age of pubertal status (year) with sleep duration (9+ hours versus <9 hours) in Hong Kong’s “Children of 1997” Birth Cohort.

	Age 11–12 OR^#^ (95% CI)	Age 13–14 OR^#^ (95% CI)	Age 17–18 OR^#^ (95% CI)
**Overall**			
Age of menarche/voice breaking		1.05 (0.98, 1.11)	1.01 (0.94, 1.09)
Onset of Breast/Genitalia	1.02 (0.96, 1.08)	1.02 (0.96, 1.09)	1.05 (0.98, 1.12)
Onset of Pubic Hair Development	1.03 (0.97, 1.10)	1.04 (0.97, 1.12)	1.02 (0.93, 1.11)
**Girls**			
Age of menarche/voice breaking		**1**.**11** (**1**.**01**, **1**.**21**)	1.06 (0.94, 1.18)
Onset of Breast/Genitalia	1.04 (0.96, 1.13)	1.02 (0.93, 1.11)	1.08 (0.99, 1.19)
Onset of Pubic Hair Development	1.06 (0.96, 1.17)	1.09 (0.98, 1.22)	1.04 (0.92, 1.18)
**Boys**			
Age of menarche/voice breaking		1.00 (0.92, 1.10)	0.97 (0.88, 1.08)
Onset of Breast/Genitalia	0.995 (0.91, 1.09)	1.03 (0.95, 1.12)	0.99 (0.89, 1.11)
Onset of Pubic Hair Development	0.996 (0.90, 1.10)	1.01 (0.92, 1.10)	0.98 (0.87, 1.11)

^*^Adjusted for parents’ place of birth, highest parental occupation, household income per head and highest parental education levels.

^#^Odds ratio (OR) per 1-year older age of puberty; thus, a significant OR >1 indicates that older age of puberty is associated with higher odds of longer sleep duration (9+ hours).

Bold font: Statistical significance.

### Mendelian randomization study

#### Genetic predictors of pubertal timing (exposure)

**Figure 1:**
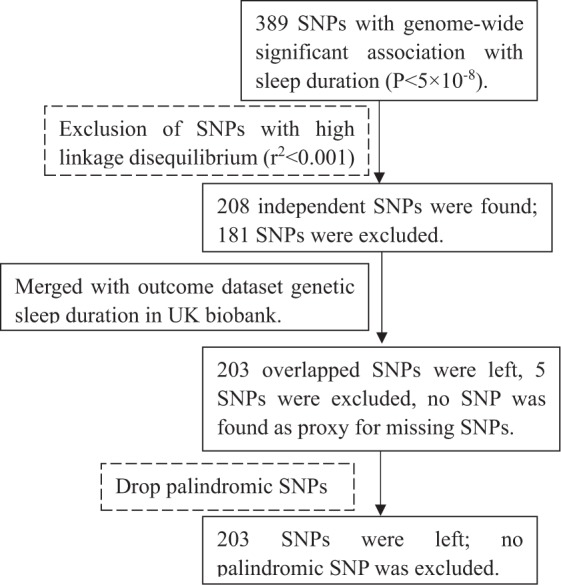
Selection of SNPs for age of menarche related to sleep duration used in Mendelian Randomization.

In total 389 single nucleotide polymorphisms (SNPs) predicting age at menarche (per year) at genome-wide significance (p-value < 5 × 10^−8^) were obtained from summary genetic associations concerning 329,345 women of European ancestry. These summary genetic associations are based on the ReproGen consortium (N = 179,117 from 40 studies), in addition to 23andMe (N = 76,831) and the UK Biobank (N = 73,397)^23^, with F statistics ranging from 29 to 953 (Appendix Table 3). The 389 loci explained 7.4% of the variance in age of menarche and the overall F-statistic was about 63. Of these 389 SNPs, 181 SNPs were excluded because of linkage disequilibrium (R^2^ < 0.001). Of the remaining 208 SNPs, 203 SNPs were available for sleep duration and no proxy was found for the 5  missing SNPs, giving 203 SNPs. Figure 1 shows the selection of SNPs related to age of menarche used as instruments. For age of voice breaking 11 genome-wide significant (p-value < 5 × 10^−8^) signals for age of voice breaking were obtained from 55,871 European men from the 23andMe study^24^ After excluding 7 correlated SNPs, 4 independent SNPs were left^24^. Two independent SNPs (p-value < 5 × 10^−6^) predicting age at Tanner stage age in girls and one in boys were obtained from 11,000 Europeans from the Early Growth Genetics (EGG) Consortium^25^. Appendix Table 3 summarizes the information extracted for each SNP.

#### Genetic associations with sleep duration (outcome)

Genetic associations with sleep duration were obtained from the UK Biobank^26^, which recruited more than 500 000 people of white British ancestry (intended age range 40–69 years) in Great Britain from 2006 to 2010.

Later age at menarche was associated with longer adult sleep duration using all methods except MR-Egger where the confidence interval included the null value. Heterogeneity was high (p < 0.0001), but the MR-Egger intercept did not indicate horizontal pleiotropy (p = 0.95). MR-PRESSO removed some SNPs as outliers (P < 0.0001), but the corrected estimates also showed a positive causal effect of age of menarche on sleep duration. The results were similar after removing 10 SNPs relevant to potential confounders and 7 potentially pleiotropic SNPs (Appendix Table 4). In boys, age of voice breaking was positively associated with sleep duration but the confidence interval included the null value. Tanner stage age in girls and boys was unrelated to sleep duration (Table 3).

**Table 3:** Mendelian Randomization Estimates of the Effect of puberty timing (year) on adult sleep duration (category).

	Puberty timing	SNPs	Average F statistic	Mendelian Randomization Method	β	95% Confidence Interval	I^2^ (p-value for heterogeneity)	MR-Egger intercept (p-value)	Outliers from MR PRESSO
Women	Age of menarche	203 (P < 5 × 10^−8^)	73.2	IVW with random-effects	**0**.**020**	**0**.**006**, **0**.**034**	46.0% (<0.0001)	0.0001 (0.95)	rs9972653 rs35935052
				WM	**0**.**021**	**0**.**003**, **0**.**040**			
				MR Egger	0.020	−0.018, 0.058			
				Corrected MR PRESSO	**0**.**017**	**0**.**003**, **0**.**030**			
	Tanner stage	2 (P < 5 × 10^−6^)	26	IVW with random-effects	0.008	−0.035, 0.051	82.9% (0.02)	NA	NA
Men	Age of voice breaking	4 (P < 5 × 10^−8^)	84.5	IVW with random-effects	0.015	−0.035, 0.064	57.2% (0.06)	0.10 (0.425)	NA
				WM	0.003	−0.034, 0.04			
				MR Egger	−0.086	−0.338, 0.166			
				Corrected MR PRESSO	0.015	−0.035, 0.064			
	Tanner stage	1 (P < 5 × 10^−6^)	25	Wald ratio method	0.005	−0.037, 0.048	NA	NA	NA

IVW: Inverse Variance Weighting

WM: Weighted median method

MR-PRESSO: Mendelian randomization pleiotropy residual sum and outlier

Bold font: Statistical significance.

## Discussion

In a population-representative Chinese birth cohort (“Children of 1997”)^22^ from an understudied non-Western population, age of menarche was positively associated with sleep duration at about 13.5 years. This finding was validated in a Mendelian Randomization study of the association of age of menarche with adult sleep duration. The evidence was not very supportive of pubertal markers causing sleep duration in boys or for other indicators of pubertal timing.

Our observational study extends previous studies by using separate indicators of the onset of puberty clinically assessed by a trained physician evaluation, rather than self-reports^19,27^. A previous genetic study using linkage disequilibrium (LD) score regression found genetic correlations between sleep duration and age of menarche^28^. Our two-sample MR study confirmed these findings with directional estimates using more genetic instruments and a larger sample. However, earlier age of voice breaking in boys was unrelated to sleep in both the observational and the MR study.

We used observational and two-sample MR designs to assess the association of earlier puberty with sleep duration, but limitations exist. First, child sleep duration was reported, which might be less accurate than actigraphy or polysomnography. However, self- and parentally-reported sleep duration has been widely used in previous studies^29^. Second, observationally sleep duration in children could only be analyzed in two groups, reducing discrimination. However, the National Sleep Foundation has suggested that 9–11 hours sleep is appropriate for 6–13 year olds^30^. Third, adjustment was not made for other factors such as diet and physical activity which may affect pubertal timing and sleep duration. However, we expected systematic rather than differential effects from residual or unmeasured confounding. Fourth, MR has three assumptions. First, strong associations of the genetic predictors with the exposure are required. In the current study, the SNPs for age of menarche and voice breaking all reached genome-wide significance with a high average F-statistic (73.2 for age of menarche and 84.5 for age of voice breaking). Some of these SNPs are in genes functionally relevant to puberty timing. For example, rare coding mutations in *MKRN3* cause central precocious puberty^31^. The *DLK1* locus confers a substantial decrease in the age of pubertal timing and *FSHB* confers earlier puberty timing through promoting higher levels of hypothalamic–pituitary–gonadal axis activity. Although we cannot rule out the possibility that some SNPs of uncertain biological function are included with corresponding risk of pleiotropy, we thoroughly investigated pleiotropic effects through multiple sensitivity analysis. Second, similar results after removing potentially confounding SNPs show the independence of the genetic variants from the confounders. Third, SNPs affecting sleep duration via mechanisms other than via puberty may generate a bias. In terms of such pleiotropy, we found no statistical evidence (from MR-Egger). However, the I^2^ statistic suggested a certain level of heterogeneity, but the weighted median and MR-Egger provided consistent positive estimates similar to those from IVW. We found some outliers from MR-PRESSO, but the main findings were not changed after excluding these outliers. Estimates were similar after removing potentially pleiotropic SNPs, again suggesting that the results were less likely to result from pleiotropy. We have adequate power to detect the observed effect size for age of menarche. However, the limited number of SNPs for voice breaking (4) and Tanner stage (2 for girls, 1 for boys) means we do not have adequate power to detect the small observed effect sizes for the other earlier puberty markers (Appendix Table 5). Replication is required when a larger study becomes possible. Fifth, the MR estimates could be confounded by population stratification, but we predominantly used genetic studies in people of European ancestry with genomic control used for both exposure and outcome, which should minimize any such bias. Sixth, we performed the observational study in a Chinese population, but the MR study was restricted to people largely of European descent, which may reduce the comparability. However, we would normally expect causal factors to act consistently in different populations unless they act by a mechanism whose relevance varies across populations^32^. We know of no reason why age of puberty should have different effects on sleep in Chinese and people of European descent. Finally, since only summary statistics, rather than individual level data, for the exposure and outcome from two different samples were used, we were unable to check for possible non-linear associations of earlier puberty with sleep duration. However, the risk of chance associations resulting from the  underlying data structure in single sample is reduced  when using  separate sample instrumental variable analysis^33^.

Although the mechanisms underlying the causal effect of earlier puberty on sleep duration are unclear, several potential explanations exist for our findings. First, sex hormones may play a role, since dramatic changes in testosterone and estrogen occur at puberty. In an RCT, estrogen increased sleep duration in postmenopausal women^34^, but the effect could differ by baseline levels. Animal studies found estrogen suppresses sleep in female rats^35,36^. As such, the mechanisms underlying the effects on sleep duration in women remain to be clarified. In men testosterone shortened sleep duration^16^. Second, earlier age of puberty was associated with higher risk of depressive symptoms only in girls in this cohort^37^, which could account for the different estimates of earlier puberty with sleep duration by sex. Third, puberty-related sex differences in hypothalamic–pituitary–adrenal (HPA) axis activity may be another contributing factor, such as via corticosterone^38^.

Age of menarche causally affects obesity^13^ and cancer^39^. Age of menarche also affecting sleep duration may explain the observed, but possibly non-causal relation of sleep duration with obesity^28^. Sex-specific associations are consistent with previous observational studies showing the association of earlier puberty with sleep duration was less clear in boys^18,27^, which may partly be due to the lack of recordable and validated measures of pubertal timing compared with the more clear-cut milestone of menarche in girls. This means some estimates in boys could be biased towards the null by non-differential misclassification. For example, in the observational study, we had a larger sample with age of menarche (n = 3,177) than other pubertal markers. However, the same direction of effect estimates in women and men in the MR study suggests a role for pubertal timing in sleep duration in general rather than menarche specifically. Together with evidence of a  similar causal effect of age of voice breaking on obesity and cardio metabolic traits^24^, our results  could indicate  sleep duration is a marker, not a driver, of obesity^40^ and possibly other chronic diseases.

## Methods

### Observational study

First, we used the “Children of 1997” birth cohort to conduct the observational study, which is a population-representative Chinese birth cohort (n = 8,327) that covered 88% of all births in Hong Kong from April 1 to May 31, 1997, described in detail elsewhere^22^. Baseline characteristics, including SEP (parental education and  an indicator of parental migrant status) and birth characteristics were obtained from a self-administered questionnaire at recruitment^41^. Passive follow-up via record linkage was instituted in 2005 to obtain pubertal stage from the Student Health Service (SHS), based on an internal reference number, Department of Health, which provides free annual check-ups for all school students. Active follow-up via direct contact was instituted in 2007, with surveys conducted in 2008/9 (Survey I), 2010/12 (Survey II), 2011/12 (Survey III) and a “Children of 1997” Biobank Clinical follow-up in 2013-6.

#### Exposure – Age of puberty

Markers of puberty including breast/genitalia, pubic hair development and age of menarche/voice breaking were the exposures. Pubertal status were visually assessed by physicians at the SHS according to the criteria of Marshall and Tanner in grades 1, 3, 5, and 7 (usually at 6–7 years, 8–9 years, 10–11 years, and 12–13 years, respectively)^42,43^. We defined the onset of puberty as onset of breast development for girls and genital development for boys, as measured by a change from Tanner stage I to stage II. Since the exact age of pubertal onset could not be precisely observed, we assumed the onset occurred midway between the latest time point when Tanner stage I was observed and the earliest time point when Tanner stage II was observed, assuming equal intervals between Tanner stages^44,45^. The age of onset of pubic hair development was estimated in same way. Children with infeasible sequences of pubertal stages, such as Tanner stage II before stage I, were excluded (n = 87)^37^. Age of menarche was self-reported at SHS clinics, in Survey III and in the “Children 1997” Biobank Clinical follow-up (for the Chinese birth cohort). Age of voice breaking was self-reported in Survey III and in the “Children 1997” Biobank Clinical follow-up.

#### Outcome – Sleep duration

The main outcome was sleep duration, which was assessed at three time points: Survey I (11.5 years), Survey II (13.5 years) and at the Biobank Clinical follow-up (17.5 years) in the Chinese “Children of 1997” birth cohort. Sleep duration was obtained in Survey I and II using a parent-reported questionnaire. Sleep duration was asked as “≤1 hours”, “2–4 hours”, “5–8 hours”, “9–12 hours” and “≥13 hours”, but almost all reported “5–8 hours” or “9–12 hours” sleep, so sleep duration was classified as <9 hours and 9+ hours. Sleep duration (in hours) was also obtained by self-report in the Biobank Clinical follow-up from the difference between bedtime and wake-up time, reported as the most common evening bedtime and wake-up time during the past month at about 17.5 years old. We also considered sleep duration as <9 hours and 9+ hours for consistency.

### Mendelian randomization

Second, in order to valid the observational results, we used summary genetic associations from 2 different genome-wide association studies (GWAS) to test each association. We obtained genetic predictors of age of menarche from a GWAS of a combined study including ReproGen, 23andMe and UK Biobank^23^, of age of voice breaking from 23andMe^24^, and of Tanner stage from EGG^25^. We obtained genetic associations with sleep duration from UK Biobank^26^, restricted to participants of European descent. We obtained SNPs strongly (p-value < 5 × 10^−8^) associated with age of puberty from the largest and most recent genome-wide association studies (GWAS)^23–25^. Linkage disequilibrium between these SNPs was identified using the “Clumping” function of MR-base^46^. We used UK Biobank data to check for any associations at Bonferroni corrected significance of the selected SNPs with potential confounders, such as education, smoking, physical activity and alcohol use^47^, using the UK Biobank, a large cohort study accessible to researchers worldwide^26^. We repeated the analysis after removing these SNPs in sensitivity analysis. Potentially pleiotropic effects (linked to the outcome other than via sleep) of the chosen SNPs were obtained from comprehensive curated genotype to phenotype cross-references, Ensembl and PhenoScanner^48^. As sensitivity analysis we also repeated the analysis after excluding potentially pleiotropic SNPs. To identify any unknown pleiotropic effects, statistically, we used MR-Egger and the Mendelian randomization pleiotropy residual sum and outlier (MR-PRESSO) test^49^.

These genetic instruments were applied to the largest publicly available GWAS of sleep duration from the UK Biobank, as described above. The GWAS includes 335,410 unrelated individuals of white British ancestry and provides sex-specific and overall genetic associations [35], adjusted, where appropriate, for sex, age, age squared, the interaction of age and sex, of sex and age squared and the first 20 principal components^47^. Sleep duration was considered in three ordered categories: <7 hours, >=7 and <8 hours and ≥8 hour.

### Statistical analysis

In the “Children of 1997” birth cohort we used chi-squared tests and Cohen effect sizes to compare confounders for children with and without information about pubertal status. The association of pubertal timing with sleep were assessed using multivariable linear regression. Confounders were selected as likely common causes of sleep duration and pubertal timing^50^, including parents’ place of birth, highest parental occupation, household income per head, highest parental education level^41^. Multicollinearity was assessed by variance inflation factor (VIF). A VIF of 10 or above suggests that interpretation of the relevant coefficients could be problematic^51^.

To account for loss to follow-up, we used a combination of multiple imputation (MI) and IPW^52^. First, we used multiple imputation to predict missing confounders and exposures^53^. Second, we estimated IPWs using logistic regression to retrieve the original sample^54^. Third, Rubin’s Rules were used to combine each IPW effect estimator with its corresponding sandwich variance estimator.

In the two-sample MR study, the causal associations of age of menarche with sleep duration were obtained using instrumental variable analysis. The F statistic for each SNP and overall was calculated to evaluate the strength of the instrument^55,56^. We pooled Wald estimates (SNP on outcome/SNP on exposure)^57^ for independent SNPs (R^2^ < 0.01) using IVW meta-analysis with multiplicative random effects which assumes balanced pleiotropy. To assess heterogeneity, we used the I^2^ statistic, where a higher value indicates more pleiotropy^58^. However, given the possibility of unknown unbalanced pleiotropy, IVW could be invalid. When an exposure had at least 3 genetic predictors, we used the weighted median (WM) and MR-Egger with more relaxed assumptions. The WM gives robust estimates as long as valid SNPs contribute >50% of the information. MR-Egger with wide confidence intervals provides valid estimates and checks for potentially unknown horizontal pleiotropy (the SNPs affect the outcomes via mechanisms other than sleep duration) through non-null intercept, but it requires that  the INstrument Strength Independent of Direct effect (INSIDE) assumption is satisfied. Since MR-Egger could not detect outliers and has limited statistical power^59^, MR-PRESSO was used to identify horizontal pleiotropic outliers and if necessary to  correct for pleiotropy via outlier removal^49^. Using 100,000 simulations, we obtained the empirical *p-*value for the MR-PRESSO global test. The Mendelian randomization estimate is valid if the global test is non-significant (*p* > 0.05). We harmonized the effect allele for exposure and outcomes on the effect allele letter for non-palindromic SNPs, and confirmed the same effect allele for palindromic SNPs, i.e., coded (A/T or C/G) from the effect allele frequency and the coding used (forward or reverse). Palindromic SNPs which could not be aligned unequivocally were replaced by proxy SNPs.

All statistical analysis was performed using Stata version 13.1 (StataCorp LP, College Station, TX) and R version 3.3.3 (R Foundation for Statistical Computing, Vienna, Austria), and the “MendelianRandomization”, “TwoSampleMR” and “MRPRESSO” packages.

### Ethics approval and consent to participate

Ethical approval for the study, including comprehensive health related analyses, was obtained from Institutional Review Board of the University of Hong Kong/Hospital Authority Hong Kong West Cluster (HKU/HA HKW IRB). The Mendelian randomization study is an analysis of publicly available summary data that does not require ethical approval.

## Supplementary information

Supplementary files.

## Data Availability

The datasets for MR analysis of this article are available from two published genome-wide association studies: https://www.nature.com/articles/ng.3841 and http://www.nealelab.is/uk-biobank. The “Children of 1997” data were available on request after approval. https://aprmay97.sph.hku.hk. The “Children of 1997” data and sample access committee is responsible for responding to birth cohort data access requests by bona fide researchers, and the relevant procedures involved (e.g., application and suggested revisions). The committee will also ensure access to the data is in line with the interests of the birth cohort participants, and access is in accordance with said protocol (i.e. measures to avoid confidentiality breaches, and notification of relevant publications arising from the requested data).
