# Supplementary material for: Age of puberty and Sleep duration: Observational and Mendelian randomization study
Source: Sci Rep. 2020 Feb 21;10:3202. doi: 10.1038/s41598-020-59811-9 (PMC7035269; doi:10.1038/s41598-020-59811-9)
Supplement: Supplementary file 1 — Supplementary files. [file 41598_2020_59811_MOESM1_ESM.docx]

**Age of puberty and Sleep duration: Observational and Mendelian randomization study.**

Jiao Wang^1^, Man Ki Kwok^1^, Shiu Lun Au Yeung^1^, Jie Zhao^1^, Albert Martin Li^2^, Hugh Simon Lam^2^, Gabriel Matthew Leung^1^, Catherine Mary Schooling^1,3*^

**Affiliations:**

^1^School of Public Health, Li Ka Shing Faculty of Medicine, The University of Hong Kong, Hong Kong, China;

^2^Department of Pediatrics, Faculty of Medicine, The Chinese University of Hong Kong, Hong Kong SAR, China;

^3^City University of New York, School of Public Health and Health Policy, New York, USA.

***Correspondence Author:** Catherine Mary Schooling

School of Public Health

Li Ka Shing Faculty of Medicine

The University of Hong Kong

G/F, Patrick Manson Building (North Wing)

7 Sassoon Road, Hong Kong SAR, China

Tel: (852) 3917 6732; Fax: (852) 3520 1945

E-mail: cms1@hku.hk

Appendix Table 1. Characteristics of Included Participants (n=4,882) and Excluded Participants (n=3,393) to puberty status in the “Children of 1997” Birth Cohort, Hong Kong

| **Characteristics** | | **Classification** | | | | | **N** | **Respondents**  **n (%)** | **Non-respondents**  **n (%)** | **P value for**  **Chi-squared** | **Cohen’s**  **effect size** |
| --- | --- | --- | --- | --- | --- | --- | --- | --- | --- | --- | --- |
| Sex | | | |  | | |  |  |  | <0.01 | 0.33 |
|  | Girl | | | | | | 3899 | 2961(60.7) | 938(27.6) |  |  |
|  | Boy | | | | | | 4340 | 1921(39.3) | 2419(71.3) |  |  |
| Parents’ birthplace | | | |  | | |  |  |  | 0.47 |  |
|  | Both parents migrant | | | | | | 1925 | 1156(24.0) | 769(25.5) |  | 0.02 |
|  | One of parent migrant | | | | | | 1626 | 980(7.3) | 646(6.1) |  |  |
|  | Both parents Hong Kong | | | | | | 4216 | 2608(53.4) | 1608(55.0) |  |  |
| Highest parental occupation | | |  | | | |  |  |  | <0.01 | 0.07 |
|  | I (professional) | | | | | | 1708 | 1046(24.7) | 662(23.5) |  |  |
|  | II (managerial) | | | | | | 1121 | 625(14.8) | 496(17.6) |  |  |
|  | IIINM (nonmanual skilled) | | | | | | 2037 | 1298(30.7) | 739(26.3) |  |  |
|  | IIIM (manual skilled) | | | | | | 1210 | 700(16.6) | 510(18.1) |  |  |
|  | IV (semi-skilled) | | | | | | 726 | 404(9.6) | 322(11.5) |  |  |
|  | V (unskilled) | | | | | | 238 | 155(3.7) | 83(3.0) |  |  |
| Household income per head in quintiles | | | | | |  |  |  |  | <0.01 | 0.06 |
|  | 1st quintile (HK$ 1751 ± 413) | | | | | | 1447 | 806(18.5) | 641(22.5) |  |  |
|  | 2nd quintile (HK$ 2856 ± 325) | | | | | | 1480 | 899(20.7) | 581(20.4) |  |  |
|  | 3rd quintile (HK$ 4362 ± 556) | | | | | | 1423 | 845(19.4) | 578(20.3) |  |  |
|  | 4th quintile (HK$ 6822 ± 886) | | | | | | 1415 | 901(20.7) | 514(18.0) |  |  |
|  | 5th quintile (HK$ 14850 ± 16050) | | | | | | 1436 | 900(20.7) | 536(18.8) |  |  |
| Highest parental education levels | | | | |  | |  |  |  | <0.01 | 0.04 |
|  | Grade 9 or below | | | | | | 2461 | 1415(29.4) | 1046(32.1) |  |  |
|  | Grade 10-11 | | | | | | 3437 | 2115(43.9) | 1322(40.6) |  |  |
|  | Grade 12 or above | | | | | | 2176 | 1286(26.7) | 890(27.3) |  |  |

*Cohen effect sizes have three levels: 0.1 for small, 0.3 for medium and 0.5 for large. Cohen effect sizes are calculated as $\sqrt{\sum\left( p0i-p1i \right)^{2}}/p0i$, where the sum is over the categories and *p0i* is the proportion in the ith category in the respondents and *p1i* is the proportion in the ith category in the non-respondents.

Appendix Table 2. The variance inflation factor (VIF) in observational study.

| **Confounders** | **VIF** |
| --- | --- |
| Parents’ birthplace | 1.43 |
| Highest parental occupation | 2.18 |
| Household income per head in quintiles | 2.38 |
| Highest parental education levels | 2.09 |

Appendix Table 3. Characteristics of SNPs used in the Mendelian Randomization study of the Effect of age of puberty on adults sleep duration.

| SNP | | Chr. | | Position (b37) | Effect allele | | | F statistic | | EAF | | BETA | | SE | | P-value | | Nearest gene | Proxy SNP | |
| --- | --- | --- | --- | --- | --- | --- | --- | --- | --- | --- | --- | --- | --- | --- | --- | --- | --- | --- | --- | --- |
| rs6678140 | | 1 | | 8436802 | t | | | 43 | | 0.673 | | -0.0269 | | 0.0041 | | 7.54E-11 | | RERE |  | |
| rs12125335 | | 1 | | 21385436 | t | | | 62 | | 0.86 | | -0.0496 | | 0.0063 | | 2.33E-15 | | EIF4G3 |  | |
| rs141847393 | | 1 | | 27212209 | t | | | 31 | | 0.918 | | 0.0396 | | 0.0071 | | 2.96E-08 | | GPN2 |  | |
| rs11209331 | | 1 | | 41456689 | t | | | 37 | | 0.571 | | 0.0238 | | 0.0039 | | 9.83E-10 | | CTPS1 |  | |
| rs11210871 | | 1 | | 44029353 | c | | | 91 | | 0.298 | | 0.04 | | 0.0042 | | 3.07E-21 | | PTPRF |  | |
| rs643428 | | 1 | | 54728858 | t | | | 30 | | 0.593 | | -0.0219 | | 0.004 | | 3.21E-08 | | SSBP3 |  | |
| rs7516763 | | 1 | | 65972550 | a | | | 37 | | 0.469 | | 0.0232 | | 0.0038 | | 1.49E-09 | | LEPR |  | |
| rs1040070 | | 1 | | 74977870 | g | | | 164 | | 0.565 | | 0.05 | | 0.0039 | | 7.01E-38 | | FPGT-TNNI3K | rs7526762 | |
| rs11165924 | | 1 | | 98375448 | a | | | 58 | | 0.677 | | 0.0312 | | 0.0041 | | 4.83E-14 | | DPYD |  | |
| rs4561063 | | 1 | | 1.03E+08 | t | | | 64 | | 0.461 | | 0.0312 | | 0.0039 | | 7.89E-16 | | OLFM3 |  | |
| rs6661100 | | 1 | | 1.51E+08 | t | | | 49 | | 0.0921 | | 0.0471 | | 0.0067 | | 2.18E-12 | | CTSK |  | |
| rs2661339 | | 1 | | 1.63E+08 | t | | | 37 | | 0.054 | | 0.0534 | | 0.0088 | | 1.34E-09 | | RGS4 |  | |
| rs157877 | | 1 | | 1.65E+08 | a | | | 211 | | 0.127 | | -0.0843 | | 0.0058 | | 2.25E-48 | | RXRG |  | |
| rs506589 | | 1 | | 1.78E+08 | t | | | 210 | | 0.803 | | 0.0695 | | 0.0048 | | 1.54E-47 | | SEC16B |  | |
| rs11240695 | | 1 | | 2.04E+08 | a | | | 57 | | 0.251 | | -0.0333 | | 0.0044 | | 3.06E-14 | | KISS1 |  | |
| rs12040029 | | 1 | | 2.13E+08 | t | | | 39 | | 0.128 | | -0.0392 | | 0.0063 | | 4.04E-10 | | RPS6KC1 |  | |
| rs62104180 | | 2 | | 466003 | a | | | 126 | | 0.0507 | | 0.1134 | | 0.0101 | | 3.98E-29 | | FAM150B |  | |
| rs7576624 | | 2 | | 625029 | t | | | 220 | | 0.826 | | -0.0741 | | 0.005 | | 4.98E-50 | | TMEM18 |  | |
| rs7587651 | | 2 | | 10368606 | t | | | 35 | | 0.373 | | -0.0238 | | 0.004 | | 4.26E-09 | | C2orf48 |  | |
| rs17390720 | | 2 | | 44952254 | c | | | 35 | | 0.735 | | 0.0259 | | 0.0044 | | 3.7E-09 | | CAMKMT |  | |
| rs111567162 | | 2 | | 56588406 | a | | | 175 | | 0.174 | | 0.0675 | | 0.0051 | | 2.49E-40 | | CCDC85A |  | |
| rs1025128 | | 2 | | 60175475 | c | | | 32 | | 0.568 | | -0.0219 | | 0.0039 | | 1.88E-08 | | MIR4432 |  | |
| rs2312205 | | 2 | | 69704941 | a | | | 36 | | 0.822 | | 0.0302 | | 0.005 | | 1.26E-09 | | AAK1 |  | |
| rs2679894 | | 2 | | 1.06E+08 | a | | | 145 | | 0.437 | | 0.0505 | | 0.0042 | | 5.37E-33 | | GPR45 |  | |
| rs2558101 | | 2 | | 1.38E+08 | a | | | 30 | | 0.72 | | -0.0236 | | 0.0043 | | 4.28E-08 | | THSD7B |  | |
| rs35935052 | | 2 | | 1.42E+08 | t | | | 65 | | 0.148 | | 0.0437 | | 0.0054 | | 5.01E-16 | | LRP1B |  | |
| rs142058842 | | 2 | | 1.57E+08 | c | | | 178 | | 0.83 | | -0.0681 | | 0.0051 | | 2.42E-40 | | NR4A2 |  | |
| rs2271758 | | 2 | | 1.73E+08 | t | | | 30 | | 0.411 | | -0.0214 | | 0.0039 | | 3.96E-08 | | SLC25A12 |  | |
| rs842567 | | 2 | | 1.84E+08 | a | | | 46 | | 0.794 | | -0.034 | | 0.005 | | 9.38E-12 | | NUP35 |  | |
| rs10931831 | | 2 | | 2E+08 | t | | | 176 | | 0.356 | | -0.0531 | | 0.004 | | 1.87E-39 | | SATB2 |  | |
| rs16841867 | | 2 | | 2.03E+08 | c | | | 58 | | 0.885 | | 0.0456 | | 0.006 | | 2.56E-14 | | NOP58 |  | |
| rs6735626 | | 2 | | 2.13E+08 | a | | | 30 | | 0.437 | | 0.0215 | | 0.0039 | | 2.96E-08 | | ERBB4 |  | |
| rs73820560 | | 3 | | 1906245 | a | | | 31 | | 0.856 | | -0.0319 | | 0.0057 | | 1.68E-08 | | CNTN4 |  | |
| rs73035994 | | 3 | | 24206463 | t | | | 61 | | 0.972 | | -0.0907 | | 0.0116 | | 5.22E-15 | | THRB |  | |
| rs1984870 | | 3 | | 24715135 | t | | | 117 | | 0.473 | | 0.0422 | | 0.0039 | | 5.61E-27 | | MIR4792 |  | |
| rs77955256 | | 3 | | 44883523 | a | | | 34 | | 0.106 | | -0.036 | | 0.0062 | | 7.1E-09 | | KIF15 |  | |
| rs115435316 | | 3 | | 49568181 | a | | | 105 | | 0.0334 | | 0.1146 | | 0.0112 | | 1.67E-24 | | DAG1 |  | |
| rs7431217 | | 3 | | 68595634 | t | | | 34 | | 0.412 | | 0.0229 | | 0.0039 | | 6.19E-09 | | FAM19A1 |  | |
| rs9758500 | | 3 | | 86910329 | a | | | 131 | | 0.375 | | -0.0457 | | 0.004 | | 1.36E-30 | | VGLL3 |  | |
| rs10934420 | | 3 | | 1.18E+08 | t | | | 206 | | 0.505 | | -0.0546 | | 0.0038 | | 1.54E-47 | | IGSF11 |  | |
| rs2461794 | | 3 | | 1.28E+08 | a | | | 63 | | 0.276 | | 0.0342 | | 0.0043 | | 2.1E-15 | | EEFSEC |  | |
| rs6439713 | | 3 | | 1.37E+08 | a | | | 40 | | 0.315 | | 0.0259 | | 0.0041 | | 2.13E-10 | | SOX14 |  | |
| rs13322435 | | 3 | | 1.57E+08 | a | | | 80 | | 0.581 | | 0.0357 | | 0.004 | | 4.13E-19 | | LOC339894 |  | |
| rs582780 | | 3 | | 1.72E+08 | a | | | 47 | | 0.582 | | 0.0267 | | 0.0039 | | 1.41E-11 | | FNDC3B |  | |
| rs2300922 | | 3 | | 1.86E+08 | t | | | 123 | | 0.414 | | 0.0432 | | 0.0039 | | 1.11E-28 | | TRA2B |  | |
| rs2108753 | | 4 | | 3266860 | t | | | 56 | | 0.565 | | 0.0284 | | 0.0038 | | 1.2E-13 | | MSANTD1 |  | |
| rs4340786 | | 4 | | 28746246 | a | | | 74 | | 0.741 | | 0.037 | | 0.0043 | | 9.25E-18 | | MIR4275 |  | |
| rs4588499 | | 4 | | 45910674 | a | | | 36 | | 0.494 | | -0.0235 | | 0.0039 | | 1.47E-09 | | GABRG1 |  | |
| rs3113862 | | 4 | | 95143122 | a | | | 91 | | 0.599 | | -0.0373 | | 0.0039 | | 9.69E-22 | | SMARCAD1 |  | |
| rs3733632 | | 4 | | 1.05E+08 | a | | | 106 | | 0.844 | | -0.0536 | | 0.0052 | | 1.04E-24 | | TACR3 |  | |
| rs17035311 | | 4 | | 1.06E+08 | a | | | 44 | | 0.854 | | 0.036 | | 0.0054 | | 2.26E-11 | | TET2 |  | |
| rs62316795 | | 4 | | 1.33E+08 | a | | | 44 | | 0.193 | | 0.0351 | | 0.0053 | | 4.98E-11 | | PCDH10 |  | |
| rs13120031 | | 4 | | 1.77E+08 | t | | | 45 | | 0.324 | | 0.0274 | | 0.0041 | | 1.57E-11 | | VEGFC |  | |
| rs10521021 | | 5 | | 35030311 | t | | | 34 | | 0.658 | | -0.024 | | 0.0041 | | 4.77E-09 | | AGXT2 |  | |
| rs7712046 | | 5 | | 43134968 | t | | | 63 | | 0.696 | | -0.0333 | | 0.0042 | | 9.42E-16 | | ZNF131 |  | |
| rs813301 | | 5 | | 52909927 | t | | | 49 | | 0.626 | | 0.0274 | | 0.0039 | | 2.84E-12 | | NDUFS4 |  | |
| rs256350 | | 5 | | 59140876 | t | | | 31 | | 0.726 | | -0.0238 | | 0.0043 | | 2.84E-08 | | PDE4D |  | |
| rs80170948 | | 5 | | 64020316 | t | | | 39 | | 0.956 | | -0.0685 | | 0.0109 | | 2.8E-10 | | SREK1IP1 |  | |
| rs13173441 | | 5 | | 77048448 | t | | | 31 | | 0.879 | | 0.0331 | | 0.0059 | | 1.86E-08 | | TBCA |  | |
| rs17085593 | | 5 | | 95630705 | c | | | 34 | | 0.684 | | 0.0246 | | 0.0042 | | 3.53E-09 | | PCSK1 |  | |
| rs654354 | | 5 | | 1.11E+08 | a | | | 36 | | 0.383 | | -0.0233 | | 0.0039 | | 2.36E-09 | | WDR36 |  | |
| rs247520 | | 5 | | 1.11E+08 | t | | | 64 | | 0.765 | | 0.0361 | | 0.0045 | | 1.99E-15 | | LOC100505678 |  | |
| rs62379978 | | 5 | | 1.34E+08 | t | | | 140 | | 0.842 | | -0.0638 | | 0.0054 | | 6.55E-32 | | PHF15 |  | |
| rs975642 | | 5 | | 1.39E+08 | t | | | 39 | | 0.494 | | -0.0245 | | 0.0039 | | 2.51E-10 | | NRG2 |  | |
| rs1428120 | | 5 | | 1.54E+08 | t | | | 43 | | 0.572 | | 0.025 | | 0.0038 | | 7.72E-11 | | GALNT10 |  | |
| rs437836 | | 5 | | 1.57E+08 | t | | | 45 | | 0.167 | | 0.035 | | 0.0052 | | 1.15E-11 | | CYFIP2 |  | |
| rs9647570 | | 5 | | 1.67E+08 | t | | | 42 | | 0.854 | | -0.0363 | | 0.0056 | | 6.26E-11 | | ODZ2 |  | |
| rs6864818 | | 5 | | 1.69E+08 | t | | | 63 | | 0.2107 | | 0.0364 | | 0.0046 | | 4.67E-15 | | SLIT3 |  | |
| rs4701140 | | 5 | | 1.79E+08 | a | | | 37 | | 0.512 | | 0.0242 | | 0.004 | | 1.95E-09 | | RUFY1 |  | |
| rs2770957 | | 5 | | 1.81E+08 | c | | | 47 | | 0.7774 | | 0.0323 | | 0.0047 | | 5.9E-12 | | TRIM41 |  | |
| rs446745 | | 6 | | 14918298 | t | | | 31 | | 0.2407 | | -0.0258 | | 0.0046 | | 2.81E-08 | | JARID2 |  | |
| rs1539310 | | 6 | | 22562485 | a | | | 29 | | 0.7568 | | 0.0244 | | 0.0045 | | 4.59E-08 | | HDGFL1 |  | |
| rs12663002 | | 6 | | 28441634 | t | | | 56 | | 0.1287 | | 0.0427 | | 0.0057 | | 6.89E-14 | | GPX6 |  | |
| rs9349203 | | 6 | | 41893323 | a | | | 108 | | 0.5463 | | -0.0395 | | 0.0038 | | 5.93E-25 | | BYSL |  | |
| rs222440 | | 6 | | 52946320 | t | | | 42 | | 0.1863 | | -0.0325 | | 0.005 | | 9.73E-11 | | FBXO9 |  | |
| rs9382676 | | 6 | | 56859084 | t | | | 66 | | 0.7767 | | 0.0373 | | 0.0046 | | 8.77E-16 | | BEND6 |  | |
| rs7753896 | | 6 | | 76347020 | a | | | 63 | | 0.3681 | | 0.031 | | 0.0039 | | 2.46E-15 | | SENP6 |  | |
| rs1414186 | | 6 | | 77713859 | t | | | 81 | | 0.205 | | -0.0432 | | 0.0048 | | 1.99E-19 | | HTR1B |  | |
| rs6931884 | | 6 | | 1E+08 | t | | | 108 | | 0.129 | | 0.0592 | | 0.0057 | | 1.64E-25 | | PRDM13 |  | |
| rs395962 | | 6 | | 1.05E+08 | t | | | 953 | | 0.3163 | | 0.1266 | | 0.0041 | | 2.3E-213 | | LIN28B |  | |
| rs4897178 | | 6 | | 1.27E+08 | t | | | 119 | | 0.5545 | | 0.0426 | | 0.0039 | | 1.02E-27 | | CENPW |  | |
| rs117530880 | | 6 | | 1.47E+08 | t | | | 31 | | 0.9703 | | -0.0658 | | 0.0118 | | 2.58E-08 | | GRM1 |  | |
| rs6911527 | | 6 | | 1.48E+08 | t | | | 36 | | 0.2275 | | 0.0269 | | 0.0045 | | 3.18E-09 | | SASH1 |  | |
| rs6933660 | | 6 | | 1.52E+08 | a | | | 70 | | 0.3173 | | -0.0343 | | 0.0041 | | 8.99E-17 | | CCDC170 |  | |
| rs910425 | | 6 | | 1.71E+08 | a | | | 31 | | 0.4483 | | -0.0223 | | 0.004 | | 1.63E-08 | | FAM120B |  | |
| rs10268051 | | 7 | | 27763590 | a | | | 31 | | 0.7769 | | 0.0249 | | 0.0045 | | 3.88E-08 | | TAX1BP1 |  | |
| rs1079866 | | 7 | | 41470093 | c | | | 161 | | 0.8632 | | -0.0711 | | 0.0056 | | 3.65E-37 | | INHBA |  | |
| rs1470750 | | 7 | | 50576648 | c | | | 33 | | 0.5923 | | -0.0223 | | 0.0039 | | 1.44E-08 | | DDC | rs12718572 | |
| rs2267812 | | 7 | | 74138121 | a | | | 72 | | 0.7951 | | 0.0417 | | 0.0049 | | 1.69E-17 | | GTF2I |  | |
| rs1030015 | | 7 | | 78139581 | t | | | 29 | | 0.5217 | | -0.0206 | | 0.0038 | | 4.45E-08 | | MAGI2 |  | |
| rs149226155 | | 7 | | 93215658 | a | | | 34 | | 0.3474 | | -0.0238 | | 0.0041 | | 5.67E-09 | | CALCR |  | |
| rs999885 | | 7 | | 99701176 | a | | | 40 | | 0.5164 | | 0.0241 | | 0.0038 | | 2.79E-10 | | AP4M1 |  | |
| rs10237306 | | 7 | | 1.22E+08 | t | | | 55 | | 0.3895 | | 0.0296 | | 0.004 | | 1.34E-13 | | CADPS2 |  | |
| rs11767400 | | 7 | | 1.22E+08 | a | | | 47 | | 0.2959 | | 0.0289 | | 0.0042 | | 5.41E-12 | | CADPS2 |  | |
| rs12707076 | | 7 | | 1.33E+08 | c | | | 47 | | 0.3844 | | 0.0273 | | 0.004 | | 4.88E-12 | | CHCHD3 |  | |
| rs7004265 | | 8 | | 1523903 | t | | | 35 | | 0.4769 | | 0.023 | | 0.0039 | | 5.12E-09 | | DLGAP2 |  | |
| rs2724961 | | 8 | | 4560227 | t | | | 146 | | 0.4682 | | -0.0459 | | 0.0038 | | 3.76E-33 | | CSMD1 |  | |
| rs6185 | | 8 | | 25280800 | c | | | 47 | | 0.7296 | | -0.0301 | | 0.0044 | | 9.48E-12 | | GNRH1 |  | |
| rs16918378 | | 8 | | 53877882 | t | | | 66 | | 0.8769 | | 0.0478 | | 0.0059 | | 9.08E-16 | | NPBWR1 |  | |
| rs56409371 | | 8 | | 53934144 | a | | | 50 | | 0.7836 | | -0.0347 | | 0.0049 | | 1.95E-12 | | NPBWR1 |  | |
| rs11786868 | | 8 | | 77653945 | c | | | 40 | | 0.837 | | 0.0321 | | 0.0051 | | 4.27E-10 | | ZFHX4 |  | |
| rs10094506 | | 8 | | 78116203 | t | | | 111 | | 0.2807 | | -0.0454 | | 0.0043 | | 2.46E-26 | | PEX2 |  | |
| rs2441873 | | 8 | | 1.05E+08 | t | | | 36 | | 0.4106 | | 0.0235 | | 0.0039 | | 1.74E-09 | | DCSTAMP |  | |
| rs2542420 | | 8 | | 1.41E+08 | t | | | 67 | | 0.5384 | | 0.0327 | | 0.004 | | 1.52E-16 | | KCNK9 | rs2615377 | |
| rs552491 | | 9 | | 1711210 | a | | | 54 | | 0.6375 | | -0.0294 | | 0.004 | | 1.11E-13 | | SMARCA2 |  | |
| rs913588 | | 9 | | 7174673 | a | | | 80 | | 0.5034 | | -0.0339 | | 0.0038 | | 6.68E-19 | | KDM4C |  | |
| rs7849973 | | 9 | | 22819576 | c | | | 36 | | 0.6547 | | 0.024 | | 0.004 | | 2.82E-09 | | FLJ35282 |  | |
| rs1329767 | | 9 | | 73798371 | a | | | 53 | | 0.3487 | | -0.0292 | | 0.004 | | 2.43E-13 | | TRPM3 |  | |
| rs7853970 | | 9 | | 86715566 | t | | | 133 | | 0.4643 | | 0.0449 | | 0.0039 | | 2.19E-30 | | RMI1 |  | |
| rs9330454 | | 9 | | 92515514 | a | | | 53 | | 0.4299 | | -0.0307 | | 0.0042 | | 1.44E-13 | | UNQ6494 |  | |
| rs11792861 | | 9 | | 1.12E+08 | a | | | 58 | | 0.7088 | | 0.032 | | 0.0042 | | 2.51E-14 | | TMEM245 |  | |
| rs7852169 | | 9 | | 1.14E+08 | c | | | 205 | | 0.9117 | | -0.0973 | | 0.0068 | | 1.82E-46 | | PTGR1 |  | |
| rs2780243 | | 9 | | 1.21E+08 | t | | | 36 | | 0.5646 | | -0.0234 | | 0.0039 | | 2.31E-09 | | TLR4 |  | |
| rs4836984 | | 9 | | 1.27E+08 | t | | | 81 | | 0.4934 | | 0.0342 | | 0.0038 | | 3.92E-19 | | NR6A1 |  | |
| rs7907759 | | 10 | | 1730008 | a | | | 110 | | 0.4709 | | 0.0409 | | 0.0039 | | 2.43E-25 | | ADARB2 |  | |
| rs1885740 | | 10 | | 10251910 | a | | | 31 | | 0.2808 | | -0.0256 | | 0.0046 | | 3.35E-08 | | SFTA1P |  | |
| rs10906395 | | 10 | | 13541008 | t | | | 36 | | 0.6109 | | -0.0233 | | 0.0039 | | 2.28E-09 | | BEND7 |  | |
| rs61846901 | | 10 | | 51056858 | t | | | 37 | | 0.3113 | | -0.0257 | | 0.0042 | | 1.21E-09 | | PARG |  | |
| rs6415872 | | 10 | | 63660689 | a | | | 37 | | 0.4931 | | 0.0236 | | 0.0039 | | 1.52E-09 | | ARID5B |  | |
| rs7072571 | | 10 | | 71380093 | a | | | 31 | | 0.7841 | | 0.0306 | | 0.0055 | | 2.69E-08 | | C10orf35 |  | |
| rs4746113 | | 10 | | 74071178 | a | | | 34 | | 0.3094 | | -0.0244 | | 0.0042 | | 8.79E-09 | | DNAJB12 |  | |
| rs77532868 | | 10 | | 88081438 | t | | | 35 | | 0.0446 | | 0.0573 | | 0.0097 | | 3.31E-09 | | GRID1 |  | |
| rs1172955 | | 10 | | 97877320 | a | | | 105 | | 0.7001 | | -0.044 | | 0.0043 | | 6.06E-25 | | ZNF518A |  | |
| rs2066323 | | 10 | | 1.05E+08 | a | | | 37 | | 0.6021 | | -0.0237 | | 0.0039 | | 1.31E-09 | | NT5C2 |  | |
| rs10400136 | | 10 | | 1.21E+08 | a | | | 44 | | 0.5627 | | -0.0259 | | 0.0039 | | 2.73E-11 | | EIF3A |  | |
| rs12571664 | | 10 | | 1.22E+08 | t | | | 58 | | 0.7998 | | 0.0367 | | 0.0048 | | 1.85E-14 | | SEC23IP |  | |
| rs7077302 | | 10 | | 1.24E+08 | c | | | 50 | | 0.0852 | | 0.0497 | | 0.007 | | 1.08E-12 | | ATE1 |  | |
| rs4576738 | | 10 | | 1.34E+08 | a | | | 38 | | 0.4455 | | 0.0266 | | 0.0043 | | 7.2E-10 | | C10orf91 |  | |
| rs16937956 | | 11 | | 8404501 | a | | | 92 | | 0.6375 | | -0.0383 | | 0.004 | | 6.38E-22 | | STK33 |  | |
| rs10832021 | | 11 | | 13324530 | a | | | 125 | | 0.7095 | | -0.047 | | 0.0042 | | 6.27E-29 | | ARNTL |  | |
| rs4359170 | | 11 | | 16596152 | a | | | 47 | | 0.673 | | 0.0282 | | 0.0041 | | 5.99E-12 | | SOX6 |  | |
| rs16917237 | | 11 | | 27702383 | t | | | 70 | | 0.2087 | | 0.0393 | | 0.0047 | | 5.7E-17 | | BDNF |  | |
| rs11031040 | | 11 | | 30317733 | t | | | 60 | | 0.8378 | | -0.0404 | | 0.0052 | | 5.1E-15 | | C11orf46 |  | |
| rs1023955 | | 11 | | 43608835 | t | | | 51 | | 0.3976 | | -0.0279 | | 0.0039 | | 7.36E-13 | | MIR129-2 |  | |
| rs953230 | | 11 | | 46064974 | a | | | 62 | | 0.7079 | | 0.033 | | 0.0042 | | 5.52E-15 | | PHF21A |  | |
| rs10750766 | | 11 | | 65473798 | a | | | 42 | | 0.7093 | | -0.0278 | | 0.0043 | | 9.96E-11 | | KAT5 |  | |
| rs4945266 | | 11 | | 78027488 | a | | | 73 | | 0.8394 | | -0.0445 | | 0.0052 | | 1.17E-17 | | GAB2 |  | |
| rs4402316 | | 11 | | 84780098 | c | | | 44 | | 0.2407 | | 0.0313 | | 0.0047 | | 3.22E-11 | | DLG2 |  | |
| rs6590889 | | 11 | | 1.01E+08 | t | | | 119 | | 0.3404 | | -0.0437 | | 0.004 | | 8.62E-28 | | TRPC6 |  | |
| rs7114175 | | 11 | | 1.23E+08 | a | | | 248 | | 0.4963 | | -0.0599 | | 0.0038 | | 1.14E-56 | | C11orf63 | rs3816621 | |
| rs77530428 | | 12 | | 17126283 | a | | | 57 | | 0.9827 | | -0.1241 | | 0.0165 | | 6.25E-14 | | SKP1P2 |  | |
| rs10842343 | | 12 | | 24579079 | a | | | 43 | | 0.6037 | | -0.0256 | | 0.0039 | | 5.78E-11 | | SOX5 |  | |
| rs7132908 | | 12 | | 50263148 | a | | | 118 | | 0.3883 | | -0.0424 | | 0.0039 | | 6.64E-27 | | FAIM2 |  | |
| rs1131017 | | 12 | | 56435929 | c | | | 34 | | 0.4174 | | 0.0229 | | 0.0039 | | 3.75E-09 | | RPS26 |  | |
| rs1148006 | | 12 | | 75978358 | a | | | 35 | | 0.2442 | | -0.0259 | | 0.0044 | | 5.81E-09 | | KRR1 |  | |
| rs7979001 | | 12 | | 97506357 | a | | | 33 | | 0.5078 | | 0.0219 | | 0.0038 | | 6.13E-09 | | NEDD1 |  | |
| rs3764002 | | 12 | | 1.09E+08 | t | | | 46 | | 0.2645 | | -0.0304 | | 0.0045 | | 1.21E-11 | | WSCD2 |  | |
| rs11065822 | | 12 | | 1.12E+08 | t | | | 36 | | 0.3725 | | 0.0258 | | 0.0043 | | 1.96E-09 | | CUX2 |  | |
| rs7133066 | | 12 | | 1.17E+08 | t | | | 34 | | 0.8504 | | 0.0334 | | 0.0057 | | 4.21E-09 | | FBXW8 |  | |
| rs9548873 | | 13 | | 40238492 | t | | | 60 | | 0.663 | | -0.0311 | | 0.004 | | 7.5E-15 | | COG6 |  | |
| rs9568123 | | 13 | | 49475780 | a | | | 30 | | 0.8478 | | -0.0291 | | 0.0053 | | 4.06E-08 | | FNDC3A |  | |
| rs4886140 | | 13 | | 59833519 | a | | | 48 | | 0.3329 | | 0.0284 | | 0.0041 | | 3.1E-12 | | DIAPH3 |  | |
| rs1925047 | | 13 | | 74600274 | a | | | 69 | | 0.3213 | | -0.0341 | | 0.0041 | | 5.7E-17 | | KLF12 |  | |
| rs11619721 | | 13 | | 1.12E+08 | t | | | 32 | | 0.0836 | | -0.0413 | | 0.0073 | | 1.33E-08 | | TEX29 |  | |
| rs9522262 | | 13 | | 1.12E+08 | c | | | 111 | | 0.4915 | | 0.0411 | | 0.0039 | | 1.69E-25 | | TEX29 | rs2026174 | |
| rs10136330 | | 14 | | 30514335 | t | | | 34 | | 0.0426 | | -0.0579 | | 0.01 | | 6.65E-09 | | PRKD1 |  | |
| rs10138913 | | 14 | | 60943106 | t | | | 187 | | 0.3062 | | 0.056 | | 0.0041 | | 1.14E-41 | | C14orf39 |  | |
| rs10143972 | | 14 | | 93850179 | t | | | 63 | | 0.8039 | | -0.0388 | | 0.0049 | | 3.05E-15 | | UNC79 |  | |
| rs941520 | | 14 | | 99709702 | a | | | 33 | | 0.4936 | | -0.0223 | | 0.0039 | | 8.99E-09 | | BCL11B |  | |
| rs12894936 | | 14 | | 1.01E+08 | t | | | 147 | | 0.2936 | | -0.0522 | | 0.0043 | | 3.73E-34 | | WDR25 |  | |
| rs7178532 | | 15 | | 23794517 | a | | | 115 | | 0.6854 | | 0.0439 | | 0.0041 | | 2.29E-26 | | MIR4508 |  | |
| rs8040272 | | 15 | | 24824016 | a | | | 51 | | 0.8678 | | 0.0441 | | 0.0062 | | 7.79E-13 | | PWRN1 |  | |
| rs34513772 | | 15 | | 40608820 | t | | | 34 | | 0.67 | | 0.0244 | | 0.0042 | | 6.2E-09 | | PLCB2 |  | |
| rs4924538 | | 15 | | 41494364 | a | | | 37 | | 0.5129 | | -0.0255 | | 0.0042 | | 1.03E-09 | | EXD1 | rs12324159 | |
| rs1435753 | | 15 | | 47925066 | t | | | 48 | | 0.6467 | | -0.0278 | | 0.004 | | 2.91E-12 | | SEMA6D |  | |
| rs10153031 | | 15 | | 67987293 | t | | | 107 | | 0.4055 | | 0.0403 | | 0.0039 | | 6.05E-25 | | MAP2K5 |  | |
| rs1971554 | | 15 | | 83406228 | t | | | 53 | | 0.2654 | | 0.032 | | 0.0044 | | 2.35E-13 | | LOC283693 |  | |
| rs12915845 | | 15 | | 89042467 | t | | | 107 | | 0.4244 | | -0.0403 | | 0.0039 | | 3.7E-25 | | DET1 |  | |
| rs758747 | | 16 | | 3627358 | t | | | 39 | | 0.2761 | | -0.0268 | | 0.0043 | | 5.56E-10 | | NLRC3 |  | |
| rs1704528 | | 16 | | 14388750 | t | | | 154 | | 0.6618 | | -0.0508 | | 0.0041 | | 1.5E-35 | | MIR193B |  | |
| rs153793 | | 16 | | 15542199 | a | | | 38 | | 0.5198 | | -0.0234 | | 0.0038 | | 9.27E-10 | | C16orf45 |  | |
| rs113388806 | | 16 | | 24804954 | a | | | 37 | | 0.9584 | | -0.0624 | | 0.0103 | | 1.26E-09 | | TNRC6A |  | |
| rs8051833 | | 16 | | 29896390 | a | | | 93 | | 0.3415 | | -0.0404 | | 0.0042 | | 9.26E-22 | | SEZ6L2 |  | |
| rs143461173 | | 16 | | 52283158 | a | | | 33 | | 0.8063 | | 0.0288 | | 0.005 | | 6.1E-09 | | LOC100505619 |  | |
| rs9972653 | | 16 | | 53814363 | t | | | 170 | | 0.4002 | | -0.0509 | | 0.0039 | | 6.47E-40 | | FTO |  | |
| rs7359336 | | 16 | | 69733460 | a | | | 197 | | 0.5789 | | -0.0534 | | 0.0038 | | 5.33E-44 | | NFAT5 |  | |
| rs142643995 | | 17 | | 2017993 | t | | | 30 | | 0.0302 | | 0.0646 | | 0.0117 | | 3.25E-08 | | SMG6 |  | |
| rs12603280 | | 17 | | 6034754 | a | | | 68 | | 0.244 | | -0.037 | | 0.0045 | | 2.66E-16 | | WSCD1 |  | |
| rs55680968 | | 17 | | 7774047 | a | | | 37 | | 0.9282 | | -0.0455 | | 0.0075 | | 1.17E-09 | | CYB5D1 |  | |
| rs9635759 | | 17 | | 49613785 | a | | | 207 | | 0.3067 | | 0.059 | | 0.0041 | | 2.78E-46 | | CA10 |  | |
| rs2787487 | | 17 | | 53209382 | c | | | 64 | | 0.6037 | | 0.0311 | | 0.0039 | | 1.62E-15 | | STXBP4 |  | |
| rs7218751 | | 17 | | 77796437 | a | | | 46 | | 0.8111 | | 0.0333 | | 0.0049 | | 1.37E-11 | | CBX4 |  | |
| rs2659007 | | 17 | | 79217478 | a | | | 62 | | 0.4646 | | -0.0306 | | 0.0039 | | 6.23E-15 | | SLC38A10 |  | |
| rs11873906 | | 18 | | 3813464 | a | | | 139 | | 0.7195 | | -0.0507 | | 0.0043 | | 2.33E-32 | | DLGAP1 |  | |
| rs8087304 | | 18 | | 31765736 | a | | | 34 | | 0.4823 | | 0.0222 | | 0.0038 | | 6.54E-09 | | NOL4 | rs11081846 | |
| rs1512238 | | 18 | | 44748467 | a | | | 200 | | 0.4207 | | -0.0537 | | 0.0038 | | 2.48E-44 | | IER3IP1 |  | |
| rs484353 | | 19 | | 7891767 | a | | | 66 | | 0.5389 | | 0.0316 | | 0.0039 | | 8.28E-16 | | EVI5L |  | |
| rs12460047 | | 19 | | 18346228 | a | | | 43 | | 0.2828 | | -0.0283 | | 0.0043 | | 6.93E-11 | | PDE4C |  | |
| rs11668587 | | 19 | | 18829770 | a | | | 64 | | 0.6653 | | -0.0329 | | 0.0041 | | 1.86E-15 | | CRTC1 |  | |
| rs29941 | | 19 | | 34309532 | a | | | 46 | | 0.3228 | | 0.0278 | | 0.0041 | | 7.85E-12 | | KCTD15 |  | |
| rs4804025 | | 19 | | 47609223 | a | | | 95 | | 0.7038 | | -0.0409 | | 0.0042 | | 3.11E-22 | | ZC3H4 |  | |
| rs2548458 | | 19 | | 49209325 | t | | | 31 | | 0.5061 | | 0.0212 | | 0.0038 | | 3.53E-08 | | FUT2 |  | |
| rs2889128 | | 19 | | 58973929 | a | | | 34 | | 0.4563 | | 0.0222 | | 0.0038 | | 7.57E-09 | | ZNF324 |  | |
| rs852061 | | 20 | | 17109159 | a | | | 88 | | 0.3645 | | -0.0365 | | 0.0039 | | 1.58E-20 | | PCSK2 |  | |
| rs111558392 | | 20 | | 20348962 | t | | | 54 | | 0.1527 | | -0.0404 | | 0.0055 | | 2.09E-13 | | INSM1 |  | |
| rs2295094 | | 20 | | 33447915 | a | | | 47 | | 0.1585 | | 0.0364 | | 0.0053 | | 5.86E-12 | | GGT7 |  | |
| rs36093651 | | 20 | | 37287102 | t | | | 65 | | 0.2378 | | 0.0371 | | 0.0046 | | 6.51E-16 | | ARHGAP40 |  | |
| rs3746619 | | 20 | | 54823805 | a | | | 47 | | 0.0862 | | 0.0475 | | 0.0069 | | 5.52E-12 | | MC3R |  | |
| rs13043968 | | 20 | | 54830983 | a | | | 44 | | 0.1109 | | -0.0413 | | 0.0062 | | 1.92E-11 | | MC3R |  | |
| rs117143374 | | 21 | | 40555561 | t | | | 78 | | 0.8642 | | -0.0502 | | 0.0057 | | 7.98E-19 | | PSMG1 |  | |
| rs151680 | | 22 | | 22273242 | t | | | 54 | | 0.524 | | 0.028 | | 0.0038 | | 2.52E-13 | | PPM1F |  | |
| rs4303811 | | 22 | | 39157755 | a | | | 35 | | 0.1244 | | -0.0353 | | 0.006 | | 3.19E-09 | | SUN2 |  | |
| rs8136272 | | 22 | | 49678782 | a | | | 83 | | 0.7277 | | 0.04 | | 0.0044 | | 6.18E-20 | | C22orf34 |  | |
| Tanner stage in female | | | | | | | | | | | | | | | | | | | | |
| rs9391253 | 6 | | 105474309 | | | t | 27 | | 0.33 | | -0.088 | | 0.017 | | 2.5E-07 | | *LIN28B* | | |  |
| rs1149336 | 1 | | 7454877 | | | t | 25 | | 0.27 | | 0.09 | | 0.018 | | 8.7E-07 | | *LIN28B* | | |  |
| Age of voice breaking | | | | | | | | | | | | | | | | | | | | |
| rs9391253 |  | | 6q16.3 | | | t | 144 | | 0.32 | | 0.12 | | 0.01 | | 8.2E-24 | | *LIN28B* | | |  |
| rs246185 |  | | 16p13.12 | | | c | 81 | | 0.33 | | 0.09 | | 0.01 | | 5.7E-14 | | *MKL2* | | |  |
| rs9408817 |  | | 9q31.2 | | | g | 49 | | 0.67 | | 0.07 | | 0.01 | | 1.6E-10 | | *TMEM38B* | | |  |
| rs142058842 |  | | 2q24.1 | | | g | 64 | | 0.17 | | 0.08 | | 0.01 | | 2.2E-08 | | *NR4A2* | | |  |
| Tanner stage in male | | | | | | | | | | | | | | | | | | | | |
| rs246185 | 16 | | 14302933 | | | t | 25 | | 0.68 | | 0.13 | | 0.026 | | 5.01E-07 | | *MKL2* | | |  |

SNP: single nucleotide polymorphism

Beta coefficient represents the change with one replicate effect allele for puberty (units are in year)

SE standard error of the estimated coefficient.

Appendix table 4. Mendelian Randomization Estimates of the Effect of puberty timing (year) on adult sleep duration after excluding potential confounders and pleiotropic SNPs.

| **Puberty timing** | **SNPs** | **Mendelian Randomization Method** | **β** | **95% Confidence Interval** | **I^2^ (p-value for heterogeneity)** | **MR-Egger intercept (p-value)** | **Outliers from MR PRESSO** |
| --- | --- | --- | --- | --- | --- | --- | --- |
| Age of menarche | 193* | IVW with random-effects | **0.021** | **0.007,0.035** | 44.2%  (<0.0001) | 0.0001  (0.90) | rs9972653 |
|  |  | WM | **0.021** | **0.002,0.040** |  |  |  |
|  |  | MR Egger | 0.023 | -0.015,0.062 |  |  |  |
|  |  | Corrected MR PRESSO | **0.018** | **0.004,0.032** |  |  |  |
|  | 186^#^ | IVW with random-effects | **0.018** | **0.004,0.032** | 43.3%  (<0.0001) | 0.0001  (0.66) | rs9972653 |
|  |  | WM | **0.021** | **0.002,0.040** |  |  |  |
|  |  | MR Egger | 0.026 | -0.012,0.065 |  |  |  |
|  |  | Corrected MR PRESSO | **0.016** | **0.002,0.030** |  |  |  |

*10 SNPs relevant to potential confounders, such as education, smoking, physical activity and alcohol use were excluded: rs1131017, rs8051833, rs2548458, rs2267812, rs11210871, rs7526762, rs2066323, rs10400136, rs10906395, rs62104180.

#7 potential pleiotropic SNPs were excluded:rs80170948 (Daytime dozing or sleeping); rs4886140 (Sleeplessness or insomnia); rs117143374 (Morning or evening person); rs12125335 (Subjects wellbeing); rs1971554 (Schizophrenia); rs941520 (Bipolar disorder); rs3764002(Nervous feelings).

Appendix table 5. The post-hoc power calculation in two-sample Mendelian randomization study.

|  |  | **SNPs** | **Power*** |
| --- | --- | --- | --- |
| Women | Age of menarche | 203 (P<5×10^-8^) | 86% |
|  | Tanner stage | 2 (P<5×10^-6^) | 0.05% |
| Men | Age of voice breaking | 4 (P<5×10^-8^) | 0.08% |
|  | Tanner stage | 1 (P<5×10^-6^) | 0.05% |

*Post-hoc power showing the power we had for each outcome given the effect size we found <http://cnsgenomics.com/shiny/mRnd/>.
